# Distinct Roles for Bacterial and Fungal Communities During the Curing of Vanilla

**DOI:** 10.3389/fmicb.2020.552388

**Published:** 2020-09-30

**Authors:** Fei Xu, Yonggan Chen, Yingying Cai, Fenglin Gu, Kejing An

**Affiliations:** ^1^Spice and Beverage Research Institute, Chinese Academy of Tropical Agricultural Sciences (CATAS), Wanning, China; ^2^National Center of Important Tropical Crops Engineering and Technology Research, Wanning, China; ^3^Hainan Provincial Engineering Research Center of Tropical Spice and Beverage Crops, Wanning, China; ^4^College of Fisheries and Life Science, Hainan Tropical Ocean University, Sanya, China; ^5^Sericulture and Agri-Food Research Institute Guangdong Academy of Agricultural Sciences/Key Laboratory of Functional Foods, Ministry of Agriculture/Guangdong Key Laboratory of Agricultural Products Processing, Guangzhou, China

**Keywords:** vanilla, curing process, microbial community, bacterial and fungal, high-throughput sequencing

## Abstract

Vanilla produces aroma after curing. There were a few reports about the possible involvement of microorganisms during the curing process. Bacterial and fungal community was analyzed to explore the distinct roles. Alpha diversity analysis indicated that the abundance and diversity of microorganisms did not increase regularly as the curing progressed. Weighted and unweighted principal coordinates analysis (PCoA) showed that the fungal community of blanching beans was significantly different from those of the vanilla beans of other stages, respectively. *Bacillus* and *Aspergillus* were the dominant genus during the curing process. Correlation analysis indicated that the bacterial and fungal structure was positively related to the vanillin formation, respectively. The study was conducive to reveal the formation of flavor components and the biosynthesis of vanillin. Furthermore, it proposed the possible curing methods of regulating the bacterial and fungal community to increase vanillin formation.

## Introduction

*Vanilla planifolia* Andrews was native to Mexico and a typical tropical orchid crop known as the “king of food and spices” (Lubinsky et al., 2008). It is one of the most important and popular aromatic plants in food, beverages, and cosmetics (Kaur and Chakraborty, 2013). Fresh vanilla beans have almost no aroma, but produce unique aroma after curing. Early research has shown that conditioning vanilla beans were sweet, vanilla, floral, prune/raisin, spicy, woody, and smoky (Ranadive et al., 2011). Traditional curing processes typically involve four steps namely: blanching, sweating, drying, and conditioning. Fresh vanilla beans were blanched by heating or freezing to destroy the cell tissue structure. Then, the blanching vanilla beans were treated under conditions of high humidity and temperature. Sweating retains a sufficiently high moisture content for the enzyme-catalyzed reaction, while allowing sufficient moisture to escape from microbial spoilage. Then, the sweating vanilla beans were further dried by sun or air to inhibit mold growth. Lastly, the drying vanilla beans were stored in a closed box for few months, and formed the unique aroma of vanilla (Mariezcurrena et al., 2008; Sreedhar et al., 2009; Rao and Ravishankar, 2010).

It was generally believed that vanillin is mainly formed by the hydrolysis of glucovanillin by β-D-glucosidase. However, a very interesting phenomenon was that vanillin continued to accumulate as the activity of β-D-glucosidase gradually decreased during the curing process (Frenkel et al., 2011). Many researches supported that the microorganisms play an important role in the formation of vanillin. Generally, vanilla beans were blanched 3–5 min in hot water or air below 80°C, removing the microorganisms. But, lots of microorganisms were not removed from the vanilla beans (Lipkin 2013; Menon and Nayeem, 2013). In addition, no special sterilization step existed in the curing process. Vanilla beans were always in contact with the external environment. Therefore, microbial growth occurred on the vanilla beans, and the growth of the microorganisms inevitably produced abundant metabolites (Podstolski et al., 2002; Pak et al., 2004). Röling et al. (2001) found that thermophilic and thermotolerant bacilli mainly associated with *Bacillus*, which could be developed under sweating for more than a week. And, the authors also observed large differences in the number of microorganisms, species composition, and the enzymatic abilities of the isolated bacteria between different batches (Röling et al., 2001). General et al. (2009) showed that the diversity of yeast increased during the curing of vanilla. Gu et al. (2017) reported that the biosynthesis of vanillin by glucose, cresol, capsaicin, and vanillyl alcohol was widely distributed in the microbial metabolism. Chen et al. (2015) indicated that the *Bacillus* colonized on vanilla beans produced β-D-glucosidase, which mediated the hydrolysis of glucovanillin. These studies have shown that microorganisms may play an important role in the formation of the vanilla flavor.

Previous study proposed that microorganisms play an important role in the formation of vanilla flavor. In this study, the vanilla beans were cured by a hot air processing method. Based on the high-throughput sequencing method, the study analyzed the microorganisms that may involve in the curing of vanilla, and systematically studied the correlation between the microorganisms and vanilla flavor. In addition, it also partly explained the continued accumulation of vanillin at a low endogenous β-D-glucosidase activity.

## Materials and Methods

### Determination of Vanillin

The extraction and determination of vanillin was carried out according to the method of Dong et al. (2014). Four grams of vacuum freeze-dried vanilla beans powder of different curing stages was thoroughly mixed with 100 ml of 70% ethanol water solution. The samples were microwaved for 20 min at 100 W. Then, the samples were filtered and made up to 100 ml after microwave extraction. Vanillin was quantified by an external standard method. HPLC conditions were as follows: Reversed phase C18 column (Zorbax, 4.6 mm × 100 mm, 3.5 μm, Agilent), injection volume: 5 μl, flow rate: 1.0 ml/min, detection wavelength: 280 nm, column temperature: 26°C, mobile phase: 20% methanol, and 80% acidified water.

### Microorganism Collection, DNA Extraction, and PCR Amplification

Vanilla beans were collected in Hainan, China, and cured by hot air processing (Gu et al., 2017). Fresh beans were blanched (70°C, 5 min), after that, they were immediately obtained as B1 samples. Then, they underwent daily sun exposure for about 6 h to be heated. The beans were packed in cotton blankets for oven sweating at 55°C for 6 h every day and cured for 6 days, they were obtained as S1 samples. Then, the beans were dried to reach a final moisture content of 30%, and they were obtained as D1 samples. Finally, the dry beans were conditioned in closed boxes at room temperature. At the 15th day of conditioning, they were acquired as F1 samples. And after conditioning (6 months), the cured beans were acquired as F2 samples. Two hundred grams (200 g) of vanilla beans at each stage were put into a bottle containing 400 ml of 0.9% NaCl salt solution. The bottle was shaken with a reciprocal shaker for 30 min. The microorganisms of each curing stage was washed from the vanilla beans, repeated three times, and mixed thoroughly. The mix was used for microbial collection. Bacteria and fungi were collected through a 0.45 μm membrane filter before preservation with liquid nitrogen and stored at −80°C.

DNA was extracted using the FastDNA spin kit for soil (Q-BIOgene, Carlsbad, CA), following the manufacturer’s instructions. The quality of the extracted DNA was examined by agarose gel electrophoresis, and the DNA was stored at −20°C until further analysis. Primer set: ITS1F (5'-CTTGGTCATTTAGAGGAAGTAA-3'; Gardes and Bruns, 1993) and ITS2R (5'-GCTGCGTTCTTCATCGATGC-3'; White et al., 1990) was selected to target the fungal region. 515F (5'-GTGCCAGCMGCCGCGG-3') and 907R (5'-CCGTCAATTCMTTTRAGTTT-3'; Xiong et al., 2012) was used to amplify target the bacterial 16S rRNA gene. Amplification reactions were conducted under the following conditions: 95°C for 2 min, followed by 25 cycles of denaturation at 95°C for 30 s, annealing at 55°C for 30 s, and extension at 72°C for 30 s, and a final extension at 72°C for 10 min. PCR products were purified using the AxyPrep DNA Gel Extraction Kit (Axygen Biosciences, Union City, CA, United States) following the manufacturer’s instructions and quantified using the QuantiFluorTM-ST (Promega, United States).

### Sequencing and Data Analysis

The DNA product was used to construct the Illumina Pair-End library and then amplicon library was paired-end sequenced (2 × 250) on an Illumina MiSeq platform (Shanghai BIOZERON Co., Ltd). The obtained raw sequence data in the Fastq format were then demultiplexed and quality-filtered using the QIIME v1.9.0. Operational taxonomic units (OTUs) were clustered using the Usearch (V7.1) based on 97% similarity and chimeric sequences were identified and removed using Uchime (V4.2.40). The sequence was defined by the Ribosomal Database Project (RDP) Classifer against the SILVA database and UNITE database with a confidence threshold of 70% (Wang et al., 2007; Quast et al., 2013).

### Statistical Analysis

Analysis of variance was determined using the SPSS 20. Differences between groups were tested using the one-way ANOVA and Duncan’s test. The difference was considered significant at *p* < 0.05. Alpha diversity of each sample was determined using the sample coverage, Chao 1 index, Shannon diversity index, and Simpson diversity index based on the Mothur version 1.30.1. Rarefaction analysis based on the Mothur version 1.30.1 was conducted to compare the richness of species in the samples with different amounts of sequencing data and, also indicated the sequencing depth of the samples (Schloss et al., 2009). Principal coordinates analysis (PCoA) was applied to study the difference of sample community composition. Analysis of similarity (ANOSIM) was conducted to identify the significant differences between the bacterial and fungal communities. To gain insight for the metabolic potential of glucovanillin, PICRUSt2 analysis was performed based on the 16S rRNA sequencing data (Langille et al., 2013). The gene family counts for each sample were derived from the KEGG ortholog (KO).

### Sequence Accession Numbers

The bacterial and fungal raw sequences data are available in the NCBI Sequence Read Archive (SRA) database under the accession number PRJNA579849 and PRJNA579861, respectively.

## Results

### Vanillin Content

**Figure 1:**
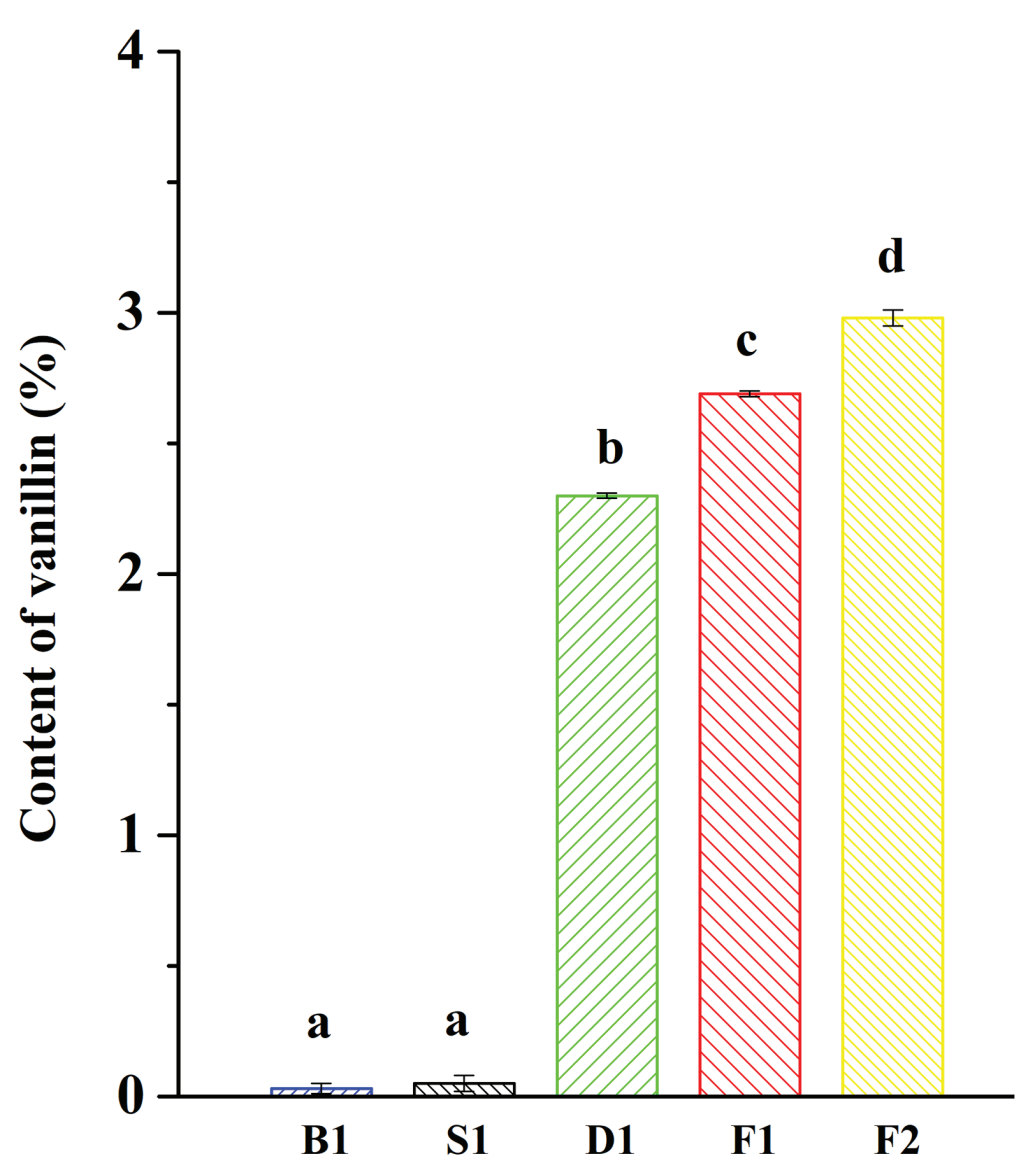
Vanillin content of vanilla beans under different curing processes. Data were subjected to Duncan’s test (*p* < 0.05). “a,” “b,” “c,” and “d” mean that the significant differences of data subjected to Duncan’s test.

The characteristic aroma of natural vanilla was composed of a large number of aromatic compounds, including the main flavor, vanillin, and more than 200 other volatile compounds (Toth et al., 2011). One of the most important indicators for measuring the quality of vanilla beans was the content of vanillin. It could be seen from Figure 1 that the vanillin content increased significantly during the progress of curing and the content of vanillin was significantly different between the different curing stages. The content of vanillin was the highest in the cured beans, reaching 2.98 ± 0.03%, while the content of vanillin was only 0.03 ± 0.02% in the blanching beans. It was reported that the content of vanillin in *Vanilla tahitensis* Moore is not higher than 1.2–1.5%, which may be attributed to the difference of origins and curing methods (Ranadive et al., 2011).

### α-Diversity

**Figure 2:**
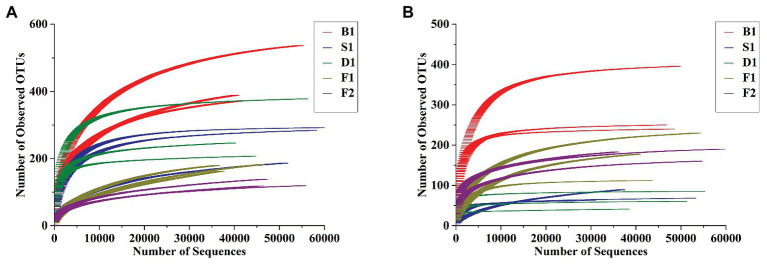
Rarefaction curves of bacterial **(A)** and fungal **(B)** communities at 97% sequence similarity level in the five curing stages of vanilla.

As can be seen from Figures 2A,B, the rarefaction curves all entered the smoothing zone in the five curing stages of vanilla, indicating that the sequencing depth can reflect the structural characteristics of the microorganisms under different curing processes. At the same time, it can be seen from Table 1, the coverage values of vanilla beans all reached above 0.99, which reflected that the sequencing results can represent the real situation of microorganisms in the vanilla beans of the different curing processes. The Chao1 index in Table 1 indicated that the total number of species of the blanching beans was the highest for both bacteria and fungi. For bacteria, the total number of species showed a downward trend in the blanching, sweating, drying, and conditioning stages. However, the total number of species was on the rise for fungi in the conditioning stages. It may be related to the ability of fungi colonized on vanilla beans to tolerate high concentrations of vanillin. Based on Shannon and Simpson indexes, it can be seen that the microbial diversity did not increase regularly (Table 1).

**Table 1:** Bacterial and fungal alpha-diversity indexes of five time-series curing of vanilla.

Microbial Community	Treatment	Coverage	Richness (Chao1)	Shannon	Simpson
Bacteria	B1	0.9985 ± 0.0004^b^	477.6667 ± 83.2666^a^	2.9600 ± 0.6780^ab^	0.1807 ± 0.1145^ab^
	S1	0.9995 ± 0.0004^a^	275.3333 ± 46.7155^b^	2.1600 ± 1.0916^bc^	0.3525 ± 0.2940^a^
	D1	0.9996 ± 0.0001^a^	288.3333 ± 86.0717^b^	3.9733 ± 0.1955^a^	0.0387 ± 0.0059^b^
	F1	0.9984 ± 0.0003^b^	235.3333 ± 22.1435^bc^	1.5067 ± 0.3213^c^	0.3355 ± 0.0941^a^
	F2	0.9993 ± 0.0002^a^	158.0000 ± 22.6495^c^	1.7533 ± 0.0586^c^	0.2965 ± 0.0090^ab^
Fungi	B1	0.9997 ± 0.0001^a^	303.6667 ± 91.3583^a^	3.3800 ± 0.2982^a^	0.1294 ± 0.0498^c^
	S1	0.9996 ± 0.0004^a^	86.6667 ± 30.6649^c^	1.4433 ± 0.8173^c^	0.4452 ± 0.2360^a^
	D1	0.9999 ± 0.0001^a^	67.6667 ± 16.0728^c^	2.3000 ± 0.3404^bc^	0.1981 ± 0.0461^bc^
	F1	0.9995 ± 0.0004^a^	188.0000 ± 69.5414^b^	1.5567 ± 0.2930^c^	0.4011 ± 0.0890^ab^
	F2	0.9995 ± 0.0003^a^	194.6667 ± 18.5831^b^	2.6800 ± 0.2858^ab^	0.1820 ± 0.0247^bc^

Values are presented as the mean ± standard deviation (*n* = 3). Means followed by the same letter for a given factor are not significantly different. *p* < 0.05.

### β-Diversity

**Figure 3:**
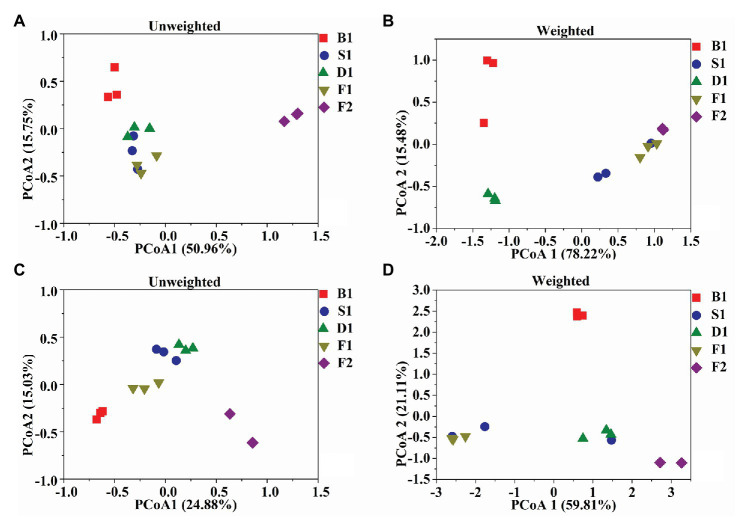
Microbial community structures in the five curing stages of vanilla. UniFrac – unweighted principle coordinate the analysis of bacterial **(A)** and fungal **(C)** community structures, UniFrac – weighted principle coordinate the analysis of bacterial **(B)** and fungal **(D)** community structures.

According to the PCoA (Figures 3A–D), the first two principal components of bacteria can account for 66.71 and 93.70% based on the Unweighted and Weighted Unifrac distances. For fungi, the first two principal components accounted for 39.91 and 80.92%. According to the ANOSIM analysis, there was a significant difference of bacteria between the blanching beans and other curing stage beans based on the Weighted Unifrac distances, and there was a significant difference between the cured beans and other curing stage beans based on Unweighted Unifrac distances. For fungi, the blanching beans and other curing stage beans were significantly different based on the Unweighted/weighted Unifrac distances. Cured beans were significantly different from the other curing stage beans based on the Unweighted Unifrac distances (Table 2).

**Table 2:** Analysis of similarity (ANOSIM) analysis of the similarity of five curing stages of vanilla.

Groups	B1 vs. S1, D1, F1, and F2	B1 vs. S1, D1, F1, and F2	F2 vs. S1, D1, F1, and B1	F2 vs. S1, D1, F1, and B1	B1 vs. S1, D1, F1, and F2	B1 vs. S1, D1, F1, and F2	F2 vs. S1, D1, F1, and B1
Sample size	15	15	15	15	15	15	15
Number of groups	5	5	5	5	5	5	5
Test statistic (R)	0.148	0.756	0.958	0.211	0.560	0.486	0.296
*p*	0.167	0.005	0.005	0.070	0.001	0.010	0.097
Number of permutations	999	999	999	999	999	999	999
Kinds of microbe	Bacteria	Bacteria	Bacteria	Bacteria	Fungi	Fungi	Fungi
Unifrac distances	Unweighted	Weighted	Unweighted	Weighted	Unweighted	Weighted	Weighted

### Bacterial Community Composition and Structure

**Figure 4:**
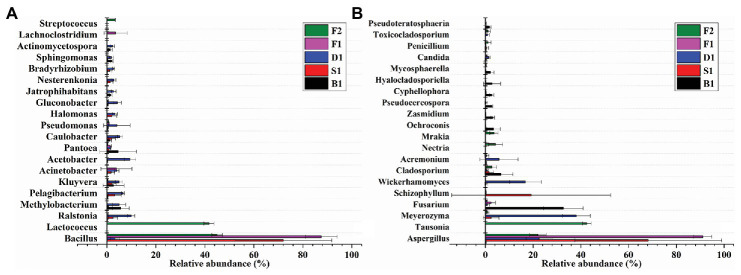
Relative abundances of 20 bacterial **(A)** and fungal **(B)** genera of the five curing stages of vanilla. Bars represent the SD of the three replicates.

*Bacillus* played a predominated role in the formation of the vanilla flavor under the curing processes (Figure 4A). Compared with the blanching beans (0.0896 ± 0.0512%), the relative abundance of *Bacillus* in the blanching stage was significantly increased to 72.0252 ± 19.7724%. Sweating may contribute to activate the *Bacillus* strains. However, the relative abundance of *Bacillus* decreased to 3.2993 ± 1.7674% in the drying stage. The relative abundance of *Bacillus* increased to 87.5184 ± 6.4880% in the conditioning stage. It was speculated that there would be new *Bacillus* colonization due to the vanilla beans exposed to the external environment for nearly 1 month, or it may be attributed to the drying resistant *Bacillus* further proliferations at room temperature. The relative abundance of *Bacillus* decreased to 44.9957 ± 2.1719% in the cured beans. It was assumed that since the conditioning was carried out under a vacuum, some aerobic *Bacillus* were unable to adapt to the changed environment, or other species increased during this stage, resulting in a decrease in the number of *Bacillus*. In addition, compared with the other curing stages, the relative abundance of *Lactococcus* in the cured beans was much greater than other curing stages and the relative abundance was 41.6964 ± 2.0479%. It was worth noting that *Streptococcus* and *Pseudomonas* also accounted for a certain proportion in the cured beans and the relative abundances were 3.3794 ± 0.0876% and 0.6907 ± 0.0402%, respectively. This indicated that *Lactococcus*, *Streptococcus*, and *Pseudomonas* could also play an important role for the conditioning of vanilla beans. Previous study showed that *Pseudomona putida* IE27 were capable of converting 150 mM isoeugenol to 16.1 g/L vanillin (Yamada et al., 2007). Furthermore, some bacterial phylum are showed in Table 3.

**Table 3:** Relative abundances of bacterial and fungal phylum of the five curing stages of vanilla.

Samples	Bacterial phylum				Fungal phylum			
	Acidobacteria	Acidobacteria	Acidobacteria	Acidobacteria	Ascomycota	Basidiomycota	Chytridiomycota	Mucoromycota
B1	0.0312 ± 0.0283	0.0312 ± 0.0283	0.0312 ± 0.0283	0.0312 ± 0.0283	0.7673 ± 0.0351	0.0289 ± 0.0168	0.0001 ± 0.0001	0.0000 ± 0.0000
S1	0.0013 ± 0.0009	0.0013 ± 0.0009	0.0013 ± 0.0009	0.0013 ± 0.0009	0.7727 ± 0.3134	0.2116 ± 0.3224	0.0000 ± 0.0000	0.0008 ± 0.0014
D1	0.0084 ± 0.0087	0.0084 ± 0.0087	0.0084 ± 0.0087	0.0084 ± 0.0087	0.9360 ± 0.0196	0.0148 ± 0.0051	0.0000 ± 0.0000	0.0000 ± 0.0000
F1	0.0002 ± 0.0001	0.0002 ± 0.0001	0.0002 ± 0.0001	0.0002 ± 0.0001	0.9644 ± 0.0097	0.0138 ± 0.0054	0.0000 ± 0.0000	0.0000 ± 0.0000
F2	0.0000 ± 0.0000	0.0000 ± 0.0000	0.0000 ± 0.0000	0.0000 ± 0.0000	0.3869 ± 0.0933	0.5067 ± 0.0108	0.0000 ± 0.0000	0.0098 ± 0.0085

### Fungal Community Composition and Structure

As can be seen from Figure 4B, *Aspergillus* dominated under the whole curing stages, which indicated that *Aspergillus* played an important role in the flavor formation of vanilla beans. Early research showed that *Aspergillus niger* could deacetylate ferulic acid into vanillic acid, and then *Pycnoporus cinnabarinus* reduced vanillic acid to vanillin (Lesage-Meessen et al., 2002). It was reported that *A. niger* could effectively release ferulic acid and caffeic acid by two different feruloyl esterase (FAEA and FAEB) in apple pomace, coffee pulp, wheat straw, corn husk, and sugar beet pulp (Benoit et al., 2006).

Compared with other curing stages, the relative abundance of *Aspergillus* was only 0.3665 ± 0.0912% in the blanching beans, while the relative abundance of *Aspergillus* in other curing stages was higher than in the blanching beans, indicating that *Aspergillus* may be involved in the formation of vanilla flavor. The relative abundance of *Aspergillus* was the highest in the conditioning stage, reaching 91.0832 ± 3.7684%. It may be attributed to the exposure of the vanilla beans to an external environment during the curing process, which made *Aspergillus* undergo further proliferation. The relative abundance of *Aspergillus* in sweating stage is 68.2543 ± 30.7098%. It was speculated that heating contributed to the activation or regeneration of *Aspergillus*. This was also supported by Röling et al. (2001), who found that the Indonesian vanilla beans were blanched (65–70°C, 2 min), resulting in significant reductions of the microbial diversity and regeneration of fungi. The abundances of *Tausonia*, *Nectria*, and *Mrakia* in the cured beans were much larger than that of other stages, which were 42.5048 ± 1.8431%, 4.2314 ± 2.9142%, and 3.5993 ± 1.7638%, respectively. This indicated that these strains may play important roles in the conditioning stage. The dominant strains of the curing stages were different in vanilla beans. Furthermore, some fungal phylum are showed in Table 3.

### Correlations Between Microbial Indicators and Vanillin

**Figure 5:**
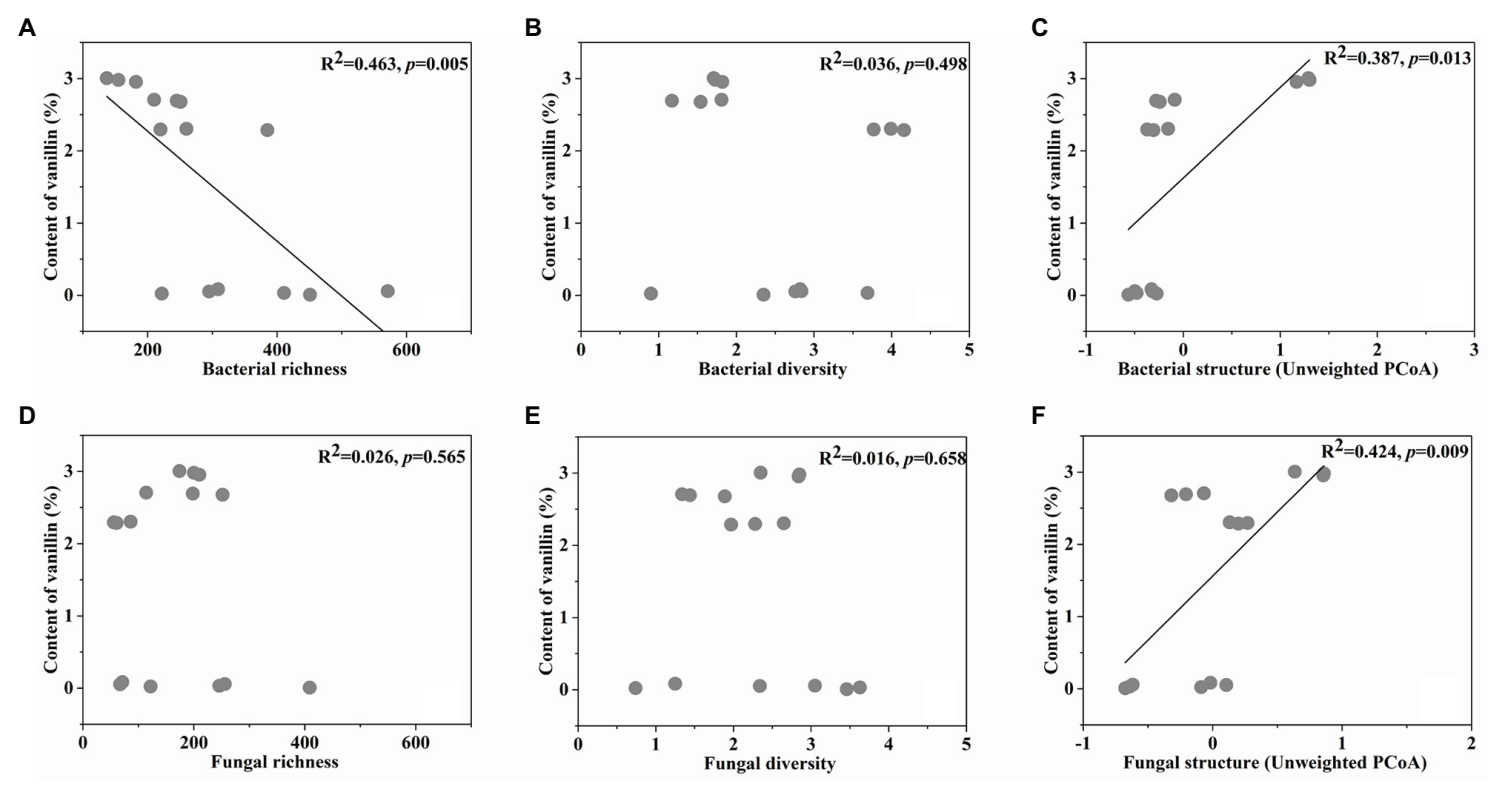
The linear regression relationship between the bacterial richness **(A)**, diversity **(B)**, structure **(C)**, fungal richness **(D)**, diversity **(E)**, structure **(F)**, and content of vanillin.

Bacterial richness, diversity, structure (Unweighted PCoA1), fungal richness, diversity, and structure (Unweighted PCoA1) were selected from the initial microbial indicators and vanillin content in the linear model, showing the best explanatory power for correlations between the microbial indicators and vanillin formation. Importantly, based on the linear regression analyses between the vanillin content and selected microbial indicators, it was shown that the unweighted bacterial structure (*R*^2^ = 0.387, *p* = 0.013), and unweighted fungal structure (*R*^2^ = 0.424, *p* = 0.009) had significantly positive correlations with vanillin content. In contrast, the bacterial richness (*R*^2^ = 0.463, *p* = 0.005) was negatively correlated with vanillin content (Figures 5A–F).

### Prediction of β-Glucosidase Genes in the Microbial Community

**Figure 6:**
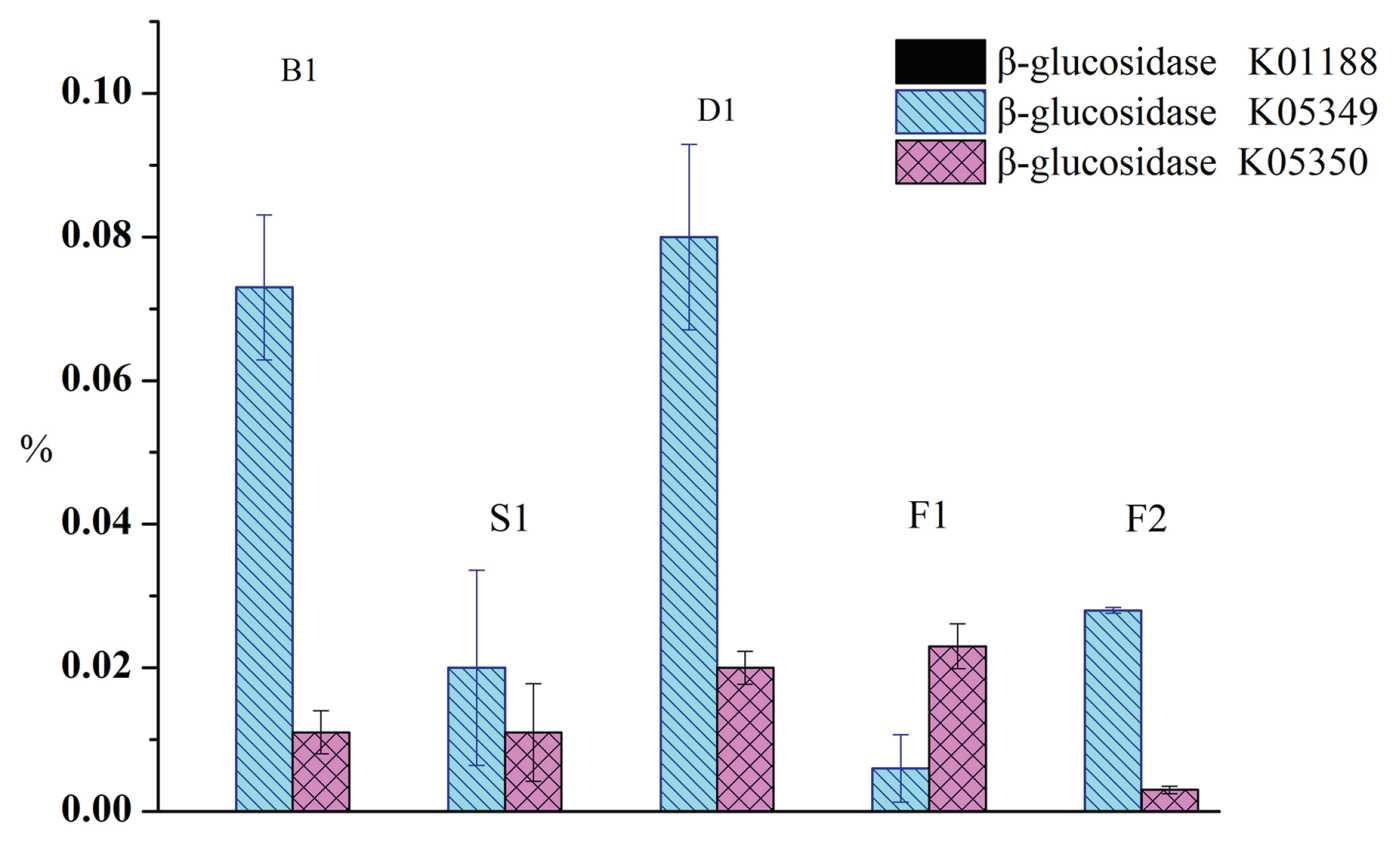
Proportion of the predicted β-glucosidase genes.

To compare the functional characteristics of glucovanillin hydrolysis during the curing process, the PICRUSt analyses was performed. Total β-glucosidase gene exceed 0.2% of the predicted genes (Figure 6). β-glucosidase gene K01188 nearly accounted 0 in all the samples. Specifically, K05349 ranged from 0.006 ± 0.0047% to 0.08 ± 0.0129% and K05350 ranged from 0.003 ± 0.0005% to 0.023 ± 0.0031%.

## Discussion

The microorganism involved in the curing of vanilla beans was supported by many researches (Röling et al., 2001; General et al., 2009; Chen et al., 2015). In this study, *Bacillus* and *Aspergillus* were found changed during the curing process. Conventional plating showed that the microbial communities mainly consisted of thermophilic and thermotolerant *Bacillus* developing under the high temperatures and maintained for over a week after scalding (Röling et al., 2001). General et al. (2009) reported that the diversity of yeast with β-D-glucosidase in vanilla contributed to the vanilla flavor development. Differently, *Aspergillus* was found to dominate under the whole curing stages in this study, which indicated that *Aspergillus* could play an important role in the flavor formation of vanilla beans. Similarly, *Aspergillus* is also found to participate in the flavor formation in Shaoxing mechanized huangjiu and katsuobushi (Liu et al., 2019; Takenaka et al., 2020).

It was found that the structure of bacteria and fungi were closely related to the vanillin content in this study. Colonizing *Bacillus* isolates produced β-D-glucosidase, which mediated the glucovanillin hydrolysis and influenced the vanillin formation (Chen et al., 2015). Furthermore, it was reported that *Bacillus siamensis* XY18 and *Bacillus subtilis* XY20 isolated from vanilla beans could increase the vanillin content (Gu et al., 2015). Additionally, fungi and yeast could convert ferulic acid into vanillin (Taira et al., 2018). Khoyratty et al. (2015) isolated the fungal endophytes from green vanilla beans and leaves in seven different areas of Reunion Island, and the fungal endophyte species of beans were different with leaves. It may be related to the vanilla flavor formed in the beans, not leaves. And, β-glucosidase genes were predicted in the microbial community of the curing process. It indicated that the isolates producing β-D-glucosidase participated in vanillin formation.

Many studies illustrated that *Bacillus* promoted the formation of the characteristic flavor compounds during fermentation in Korean soy sauce, broccoli, and cocoa beans (Paramithiotis et al., 2010; Nam et al., 2012; de Melo Pereira et al., 2013). *Aspergillus* also played an important role in the formation of flavor during the fermentation of balsamic vinegar and liquor (Wang et al., 2016, 2018). Vanilla beans were usually blanched for 3–5 min in hot water or air, and the microorganism would be changed after blanching, for example, some strains with a low thermotolerance would be removed or inhibited (Röling et al., 2001; Frenkel et al., 2011). However, some heat-resistant microorganisms would survive. The cell structure was destroyed and many glycosides seeped out after blanching, it may be more likely to promote microbial growth (Mariezcurrena et al., 2008). In addition, the vanilla beans were always in contact with the external environment during the curing process, and the microorganisms may be colonized again. The changed environment may influence the microbial structure, which may result in different flavor compounds. Ranadive et al. (2011) showed that the same variety of vanilla from different parts of the world could exhibit different flavors. It has been reported that although vanilla bean used the same curing procedure, the flavors were different in the vanilla beans from various areas of Reunion Island (Khoyratty et al., 2018). Therefore, vanilla beans cured by different methods would have the typical microorganisms during the curing process, which may induce a different flavor in vanilla beans (Zhang and Mueller, 2012; Takahashi et al., 2013).

The paper indicated that the microorganisms on vanilla beans underwent significant changes during the curing stages, especially *Bacillus* and *Aspergillus* which participated in the process. Vanilla beans under *Bacillus* – assisted vanilla curing and conventional curing produced more vanillin than those under the non-microorganism – assisted curing. Moreover, β-D-glucosidase-produced *Bacillus* isolates could be used to increase vanillin production without generating any unpleasant sensory attribute. This study provided another way to regulate the structure of microbiota used for increasing vanillin formation.

## Conclusion

The microorganisms involved in the curing of vanilla beans have been analyzed, which was conducive to a further development of the curing methods. This study explored the changed colonizing microorganisms on vanilla beans during the curing process. In the future, separating the dominant strains and carrying out the auxiliary curing of vanilla were a prominent study in our lab. Furthermore, the definitive contribution microorganisms to flavor compounds are still unknown, and it is also necessary for future study.

## Data Availability Statement

All datasets presented in this study are included in the article/supplementary material.

## Author Contributions

YCh: software. FX, YCa, and YCh: formal analysis. FX and YCa: investigation. YCa and FX: writing – original draft preparation. FG: writing – review and editing. FG: supervision. FG and FX: project administration, and FG and FX: funding acquisition. All authors contributed to the article and approved the submitted version.

### Conflict of Interest

The authors declare that the research was conducted in the absence of any commercial or financial relationships that could be construed as a potential conflict of interest.
